# From Dipolar Interactions to Tissue Heating: A Multiscale Model for Magnetic Hyperthermia

**DOI:** 10.3390/nano16171069

**Published:** 2026-08-27

**Authors:** Viorica Monica Moisiuc, Iordana Astefanoaei, Alexandru Stancu

**Affiliations:** Faculty of Physics, Alexandru Ioan Cuza University of Iasi, 700506 Iasi, Romania; monica_moisiuc@yahoo.com (V.M.M.); alstancu@uaic.ro (A.S.)

**Keywords:** magnetic hyperthermia, magnetic nanoparticles, dipole–dipole magnetic interaction, specific absorption rate (SAR)

## Abstract

Magnetic hyperthermia is a promising therapeutic technique in which magnetic nanoparticles (MNPs) generate heat when exposed to a high-frequency alternating magnetic field. The magnetic dipolar interactions between magnetic nanoparticles play an important role in the relaxation dynamics and overall magnetic heating efficiency. In this work, the thermal response of a tumoral tissue was studied considering the dipole–dipole interactions in chain-like nanoparticle assemblies. A 3D space–time model implemented in COMSOL Multiphysics 6.2 is used to investigate the temperature field and thermal damage in tumoral tissue considering magnetic relaxation mechanisms for both (i) parallel and (ii) perpendicular anisotropy configurations with respect to the applied magnetic field. Dipole–dipole interactions significantly modify the effective energy barriers involved in magnetic relaxation mechanisms, when nanoparticles are closely spaced. Interparticle spacing and MNP size are two very important parameters that influence the effective anisotropy barrier and, implicitly, the heating efficiency of magnetic nanoparticles. Moderate dipolar interactions lead to optimal SAR values, while strong interactions reduce heating efficiency due to magnetic locking. This study provides guidelines for the design of magnetic nanoparticles for hyperthermia applications.

## 1. Introduction

Magnetic hyperthermia is an innovative approach for destroying malignant/cancerous cells which uses the therapeutic heat given by the magnetic nanoparticles when an external magnetic field is applied [1,2,3,4]. The heating efficiency of magnetic nanoparticles strongly depends on their magnetic relaxation dynamics and on dipolar interactions arising in nanoparticle aggregates [5,6,7,8,9,10,11,12]. These magnetic systems absorb energy from a high-frequency electromagnetic field and convert it into heat. The therapeutic temperature values corresponding to the hyperthermic range 40–45 °C are achieved by a fine control of the magnetic field parameters (frequency and amplitude) [13,14,15,16,17,18,19,20,21,22,23]. The magnetic and thermal properties of these magnetic systems injected within tumoral tissues play an important role in the efficiency of this therapy method. Rosensweig linear response theory considers noninteracting nanoparticles and their ideal dispersion in a ferrofluid [24]. In realistic biological environments such as inside tumoral cells, MNPs tend to form aggregates in chains and/or clusters due to magnetic dipole–dipole interactions. Previous experimental and theoretical studies have shown that dipolar interactions may either increase or decrease the effective energy barrier depending on the spatial arrangement of nanoparticles and the orientation of their magnetic moments. Dormann and Mørup models describe MNP ensembles with randomly oriented anisotropy axes. They study how dipole–dipole interactions between MNPs modify the effective energy barrier for magnetization reversal and the blocking temperature through collective interaction effects [5,6,7]. These models do not assume aligned anisotropy axes. Analytical and semi-analytical models for 1D chains of MNPs were developed to understand how dipole–dipole coupling modifies the anisotropy barrier. For example, Branquinho et al. [9] proposed a linear-response model for ideal chains and showed that dipolar coupling adds an additional term to the anisotropy energy. In their model, dipolar interactions increase the effective energy barrier, shifting the optimum particle size for magnetic heating toward smaller values under strong coupling.

This paper investigates the influence of dipolar interactions in chain-like magnetic nanoparticle assemblies on the temperature field generated in tumor tissues under an applied high-frequency electromagnetic field. The novelty of the present study lies in the analysis of dipolar interaction effects, magnetic relaxation regimes, SAR generation, and the resulting thermal response in tumor tissues, providing a multiscale description linking nanoparticle interactions with macroscopic heating efficiency. Specifically, this theoretical and computational study combines an analytical description of dipole–dipole interactions in chain-like assemblies of magnetic nanoparticles with magnetic relaxation modeling based on the Néel and Brownian mechanisms. The resulting effective relaxation time is used to calculate the magnetic susceptibility, power dissipation, and specific absorption rate (SAR). These quantities are subsequently introduced as the magnetic heat source in a 3D bioheat model implemented in COMSOL Multiphysics to calculate the 3D space–time temperature distribution and thermal damage in tumoral tissues. This approach provides a direct link between nanoparticle interaction physics and the macroscopic thermal response of biological tissues.

## 2. Theoretical and Computational Model

**Figure 1:**
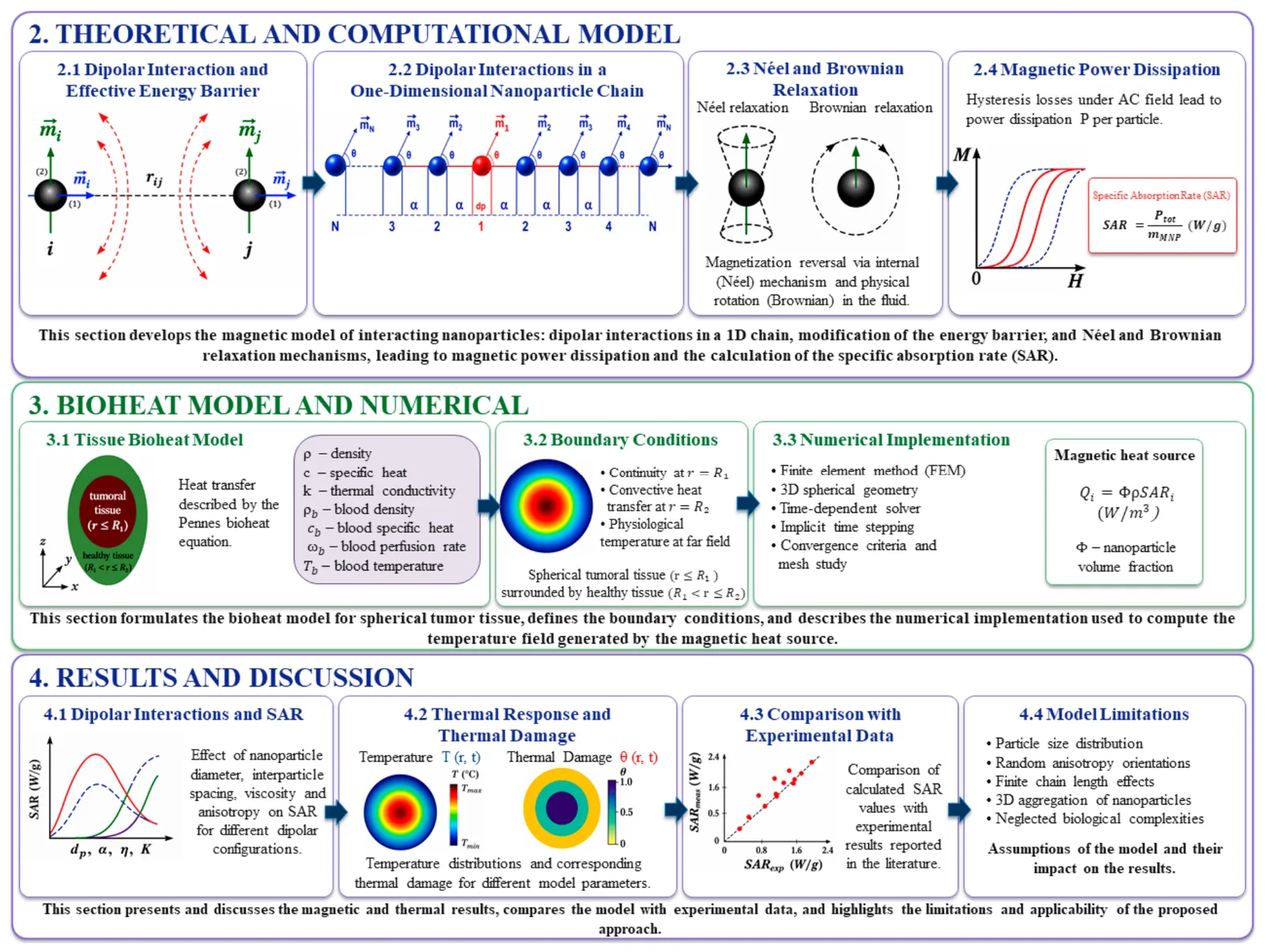
Schematic representation of this study.

This section presents the theoretical framework used to describe the influence of dipolar interactions on the effective anisotropy energy barrier of magnetic nanoparticles. The resulting effective energy barriers are subsequently introduced into the Néel relaxation time and used to calculate the specific absorption rate (SAR) employed in the thermal model. The model assumes identical single-domain superparamagnetic nanoparticles with uniaxial anisotropy arranged in ideal chain-like assemblies. Only magnetic dipole–dipole interactions are considered, while exchange interactions and particle-size dispersion are neglected (Figure 1).

### 2.1. Dipolar Interaction and Effective Energy Barrier

For two identical magnetic nanoparticles with the magnetic moments ${\vec{m}}_{1}$ and ${\vec{m}}_{2}$ ($\left|{\vec{m}}_{1}\right|\;=\;\left|{\vec{m}}_{2}\right|\;=\;m$) separated by a center-to-center distance d, the dipole–dipole interaction energy is given by [9]:

(1) $$E_{int}\;=\;\frac{\mu_{0}}{4\pi d^{3}}\left[{\vec{m}}_{1}\cdot {\;\vec{m}}_{2}-3\left({\vec{m}}_{1}\cdot \hat{\;r}\right)\left({\vec{m}}_{2}\cdot \hat{\;r}\right)\right]$$

where $\hat{r}\;=\;\frac{\vec{r}}{d}$ is the unit vector connecting the particle centers. The remaining parameters are defined in Table 1. Dipolar interactions modify the energy landscape governing magnetization reversal and, depending on the relative orientation of the magnetic moments and the interparticle axis, may either increase or decrease the effective anisotropy barrier. For a nanoparticle with uniaxial anisotropy, the effective energy barrier can therefore be expressed as

(2) $$E_{\tau }^{eff}=KV+\Delta E_{int}$$

where: *KV* is the intrinsic anisotropy barrier and $\Delta E_{int}$ represents the dipolar contribution.

In this paper, two anisotropy configurations are considered depending on the orientation of the magnetic moments: (i) parallel and (ii) perpendicular anisotropy. In the parallel anisotropy configuration, the magnetic moments (or easy axes) are aligned along the interparticle axis. In the perpendicular anisotropy configuration, the magnetic moments are oriented perpendicular to the interparticle axis.

For magnetic moments aligned parallel to the interparticle axis (${\vec{m}}_{1}∥{\vec{m}}_{2}∥\hat{r})$, the dipolar interaction increases the effective energy barrier:

(3) $${\left(E_{\tau }^{eff}\right)}_{1}\;=\;KV\;+\;3C$$

whereas for magnetic moments oriented perpendicular to the interparticle axis (${\vec{m}}_{1}∥{\vec{m}}_{2}⊥\hat{r}$), dipolar contribution reduces the barrier.

(4) $${\left(E_{\tau }^{eff}\right)}_{2}=KV-3C$$

with

(5) $$C=\frac{\mu_{0}m^{2}}{4\pi d^{3}}$$

The parallel and perpendicular anisotropy configurations considered here represent two limiting cases of the orientation-dependent dipolar interaction rather than a complete description of realistic nanoparticle assemblies. In the parallel configuration, dipolar coupling increases the effective energy barrier, whereas in the perpendicular configuration it decreases it. Arbitrary orientations are expected to generate intermediate and distributed dipolar contributions, leading to a distribution of effective energy barriers and relaxation times. Thus, the two limiting configurations provide a useful framework for establishing the range of possible effects of dipolar coupling on magnetic relaxation and SAR, while a complete description of disordered nanoparticle assemblies would require averaging over the orientational distribution.

### 2.2. Dipolar Interaction in One-Dimensional Nanoparticle Chain

**Figure 2:**
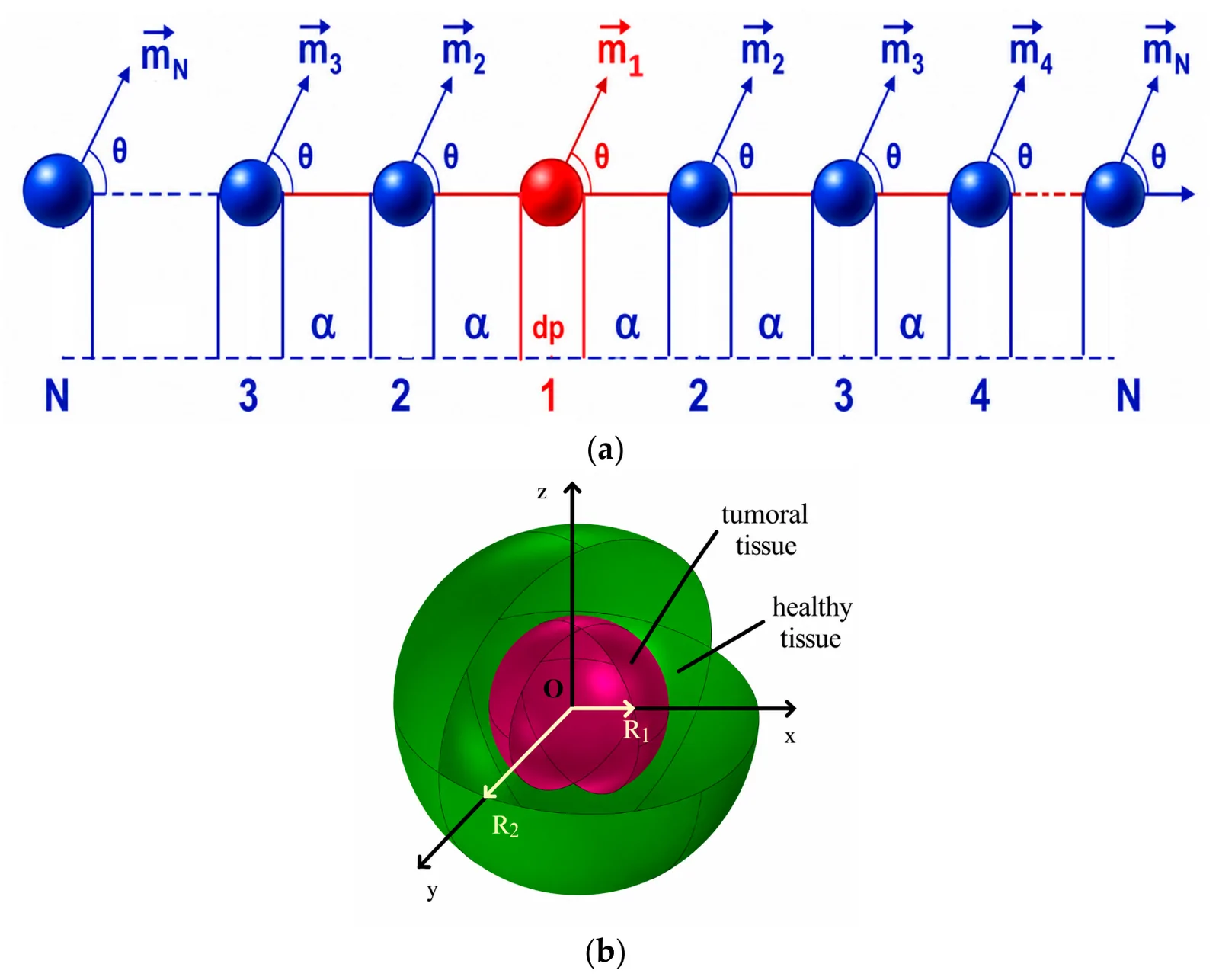
(**a**) One-dimensional chain model consisting of 2N interacting nanoparticles. (**b**) Geometric configuration of the tumoral tissue surrounded by the healthy tissue.

We consider a sufficiently long linear chain of identical superparamagnetic nanoparticles, separated by a center-to-center distance d. Each magnetic moment forms the same angle θ with the chain axis (Figure 2a). The dipolar interaction energy between two magnetic moments $m_{i}$ and $m_{j}$ separated by a distance $r_{ij\;}\;=\;nd$ can be written as:

(6) $$E_{ij}\;=\;\frac{\mu_{0}m^{2}}{4\pi \;{\left(nd\right)}^{3}}\left(1\;-\;3{cos}^{2}\theta \right)$$

Considering the dipolar contributions from neighboring particles on both sides of a reference particle, the interaction energy per particle becomes

(7) $$E_{int}\left(\theta \right)=2C\;\zeta (3)\left(1-3{cos}^{2}\theta \right)$$

where $\zeta \left(3\right)\;=\;\sum_{n=1}^{\infty }\frac{1}{n^{3}}$ is a Riemann zeta function. For a sufficiently long chain edge effects can be neglected and the dipolar sum converges to $\zeta \left(3\right)\;≅\;1.202$. The total magnetic energy per particle is therefore:

(8) $$E\left(\theta \right)\;=\;KV{sin}^{2}\theta \;+\;2C\;\zeta \left(3\right)\left(1\;-\;3{cos}^{2}\theta \right).$$

For the parallel anisotropy configuration, the effective energy barrier (8) becomes:

(9) $${\left(E_{\tau }^{eff}\right)}_{1}\;=\;KV\;+\;\beta_{1}\frac{\mu_{0}{M_{s}^{2}V}^{2}}{4\pi {\left(d_{p}\;+\;\alpha \right)}^{3}}$$

with: $\beta_{1}\;=\;+6\zeta \left(3\right)$ and ${d\;=\;d}_{p}\;+\;\alpha .$ Thus, dipolar interaction increases the effective energy barrier and stabilizes the magnetic configuration. For the perpendicular anisotropy configuration, the effective energy barrier (8) becomes:

(10) $${\left(E_{\tau }^{eff}\right)}_{2}\;=\;KV\;+\;\beta_{2}\frac{\mu_{0}{M_{s}^{2}V}^{2}}{4\pi {\left(d_{p}\;+\;\alpha \right)}^{3}},$$

with: $\beta_{2}\;=\;-6\zeta \left(3\right)$. In this configuration dipolar interactions reduce the effective energy barrier, particularly at small interparticle spacing where the dipolar coupling becomes stronger. The two configurations can therefore be written in the general form:

(11) $${\left(E_{\tau }^{eff}\right)}_{i}\;=\;KV+\beta_{i}\frac{\mu_{0}{M_{s}^{2}V}^{2}}{4\pi {\left(d_{p}\;+\;\alpha \right)}^{3}}\;i(=1,\;2)$$

where: *i* = 1 corresponds to the parallel anisotropy configuration which increases the effective energy barrier and *i* = 2 corresponds to the perpendicular anisotropy configuration in the interaction model which reduces the energy barrier. The effective energy barrier thus depends strongly on nanoparticle size $d_{p}(nm)$, interparticle spacing α(nm) and magnetic-moment orientation. Since the Néel relaxation time depends exponentially on this barrier, dipolar interactions can significantly modify the magnetic relaxation dynamics and consequently the heating efficiency of interacting nanoparticle assemblies.

### 2.3. Néel and Brownian Relaxation

MNPs are essential for controlling the temperature and the resulting thermal damage in tumor tissue during hyperthermia therapy. The efficiency of this technique strongly depends on the magnetic and thermal properties of the nanoparticles, which are determined by particle characteristics such as size, magnetic anisotropy, material type, and concentration. Understanding the relationship between these parameters and the heating efficiency is therefore essential for optimizing hyperthermia treatments.

Magnetic energy dissipation in nanoparticle systems occurs through magnetic relaxation processes. In the Néel relaxation mechanism, the magnetic moment of a superparamagnetic nanoparticle reverses its orientation across an anisotropy energy barrier due to thermal fluctuations ($k_{B}T$). In contrast, Brownian relaxation corresponds to the physical rotation of the entire nanoparticle within the surrounding fluid, allowing the magnetic moment to align with the external magnetic field. During Brownian relaxation the particle experiences viscous friction with the carrier liquid or biological medium. The energy dissipated through this friction is converted into heat, contributing to the specific absorption rate (*SAR*) defined as (${SAR}_{i}\;=\;P_{i}/\rho )$, where the parameters are defined in Table 1. The relative contribution of Néel and Brownian relaxation depends strongly on the magnetic anisotropy and on the viscosity of the surrounding medium. When the effective anisotropy barrier is low, the Néel relaxation time is short and Néel relaxation is dominant. Conversely, as the anisotropy barrier increases, the Néel relaxation time increases and internal magnetization reversal becomes progressively suppressed. In this case Brownian relaxation may become more important if the nanoparticle is free to rotate.

The relaxation dynamics of the magnetic moment is governed by the effective energy barrier (11) ${\left(E_{\tau }^{eff}\right)}_{i}$, (i = 1, 2), which depends strongly on the nanoparticle diameter $d_{p}$, the magnetic anisotropy constant K and the dipole–dipole interactions between particles. In this analysis, three physical relaxation regimes of the Néel–Brown relaxation depending on the dimensionless parameter

(12) $$\sigma_{i}=\frac{{\left(E_{\tau }^{eff}\right)}_{i}}{k_{B}T},i=1,\;2$$

are considered: (i) **regime I**—fast relaxation (superparamagnetic regime), (ii) **regime II**—intermediate (Néel–Brown contribution) and (iii) **regime III**—slow relaxation (blocked regime) [15,16].

**Regime I** corresponds to a low anisotropy barrier ($\sigma_{i}\;<\;1$), where the magnetic moment fluctuates freely and the relaxation is not thermally activated. In this case the Néel relaxation time approaches the attempt time $\tau_{N}=\tau_{0\;}\sim {\;10}^{-9}\;s$.

**Regime II** ($1\;<\;\sigma_{i}\;<\;5$) describes the transition between superparamagnetic and blocked behavior. In this region thermal energy and the effective anisotropy barrier are comparable and both Néel and Brownian mechanisms contribute to the relaxation process.

**Regime III** $\left(\sigma_{i}\;>\;5\right)$ corresponds to nanoparticles approaching the blocked state. In this case the effective barrier is high and the Néel relaxation time becomes very large $\left(\tau_{N}\;\gg \;\tau_{0\;}\right)$.

Dipolar interactions between nanoparticles modify the effective energy barrier and therefore influence the relaxation regimes. In the interaction model (*i* = 1), with parallel anisotropy configuration, dipole–dipole interactions between nearby particles oriented along the dipolar coupling direction increase the effective energy barrier, leading to:

(13a) $$\sigma_{1}\;=\;\frac{{\left(E_{\tau }^{eff}\right)}_{1}}{k_{B}T}\;=\;\frac{KV}{k_{B}T}\;+\;\beta_{1}\frac{\mu_{0}M_{s}^{2}}{24\;k_{B}T}\frac{d_{p}^{6}\;}{{\left(d_{p}\;+\;\alpha \right)}^{3}}$$

In contrast, in the interaction model (*i* = 2) with perpendicular anisotropy configuration, the magnetic moments are oriented perpendicular to the dipolar interaction direction. In this case the dipolar coupling reduces the effective anisotropy barrier and the corresponding parameter becomes:

(13b) $$\sigma_{2}\;=\;\frac{{\left(E_{\tau }^{eff}\right)}_{2}}{k_{B}T}\;=\;\frac{KV}{k_{B}T}\;+\;\beta_{2}\frac{\mu_{0}M_{s}^{2}}{24\;k_{B}T}\frac{d_{p}^{6}\;}{{\left(d_{p}\;+\;\alpha \right)}^{3}}$$

The effective relaxation time $\tau_{i}\;(i\;=\;1,\;2)$

(14) $$\tau_{i}=\frac{{\left(\tau_{N}\right)}_{i}\tau_{B}}{{\left(\tau_{N}\right)}_{i\;}{+\tau }_{B}}$$

contains the Néel relaxation time ${\left(\tau_{N}\right)}_{i}$ [15]

(15) $${\left(\tau_{N}\right)}_{i}=\left\{\begin{array}{c} \tau_{0},\;\;\;\;\;\;\;\;\;\;\;\;\;\;\;\;\;\;\;\;\;\;\;\;\;\;\;\sigma_{i}<1\;\;\\ \tau_{0}\exp\left(\sigma_{i}\right),\;1<\sigma_{i}<5\;\\ \tau_{0}\frac{\sqrt{\pi }}{2}\frac{\exp\left(\sigma_{i}\right)}{\sqrt{\sigma_{i}}},\;\;\;{\;\;\;\;\sigma }_{i}>5 \end{array}\right.\;i=1,\;2$$

and Brown relaxation time:

(16) $$\tau_{B}\;=\;\frac{3\eta V_{H}}{k_{B}T}\;$$

$V_{H}\;=\;V{\left(1\;+\;\frac{\delta }{R}\right)}^{3}$. The parameters appearing in Equation (16) are defined in Table 1.

### 2.4. Magnetic Power Dissipation

For both cases, the volumetric heating rate, developed by a magnetite particle, $P_{i}(W/m^{3})$ (i = 1, 2) in a magnetic field is given by the following relation [15,16]:

(17) $$P_{i}=\mu_{0}f\pi \chi_{i}^{\prime \prime }H^{2}$$

The magnetic susceptibility $\chi_{i}^{\prime \prime }$ is given by the following relation:

(18) $$\chi_{i}^{\prime \prime }=\frac{\mu_{0}M_{s}^{2}V}{3k_{B}T}\frac{2\pi f\tau_{i}}{1+{\left(2\pi f\tau_{i}\right)}^{2}}.\;$$

The thermal sources which are used in the modeling of heating processes in the tumoral and surrounding healthy tissues are given by the following relations: $Q_{i}\;=\;\Phi P_{i}$ (i = 1, 2). The maximum SAR values are obtained when the effective magnetic relaxation time becomes comparable to the period of the applied alternating magnetic field, i.e., when $2\pi f\tau_{i}\;≅\;1$ which corresponds to the optimal matching between magnetic relaxation dynamics and field oscillation.

The effective energy barrier modifies the Néel relaxation time, which directly affects the magnetic susceptibility and therefore the SAR. In chain-like aggregates the Brownian rotation may be partially suppressed, however, its contribution is still considered in the model for completeness.

## 3. Bioheat Model and Numerical Implementation

### 3.1. Tissue Bioheat Model

The tumoral tissue surrounded by healthy tissue is represented by a geometric configuration of two concentric tissues having the radiuses R_1_ and R_2_ (Figure 2b). Different physical processes such as conduction, convection, blood perfusion and metabolic heat generation occur within heating process of the living tissues.

The temperature developed in tissues, $T_{k}\;(k=1,2)$, is described by the bioheat transport equation [13]:

(19) $$\rho_{k}c_{k}\frac{\partial T_{k}}{\partial t}=\nabla \left(k_{k}{\nabla T}_{k}\right)+\rho_{b}{\omega_{b}}^{k}c_{b}\left(T_{a}-T_{k}\right)+Q_{met}^{k}+{\mathrm{Source}}_{k}$$

The solution of these equations describes the evolution of the space–time temperature in the geometric configuration of tumoral and surrounding healthy tissues. In Equation (19) the index k=1 represents tumoral tissue and the index k = 2 represents healthy tissue. The thermophysical parameters and their numerical values are summarized in Table 2.

$${\mathrm{Source}}_{k}=\left\{\begin{array}{l} Q_{i}\;=\Phi P_{i}=\Phi \rho {SAR}_{i}\;\;\;\;\;(i=1,\;2),\;\;\mathrm{tumoral}\;\mathrm{tissue}\;(k=1)\\ \;\;\;0,\;\;\;\;\;\;\;\;\;\;\;\;\;\;\;\;\;\;\;\;\;\;\;\;\;\;\;\;\;\;\;\;\;\;\;\;\;\;\;\;\;\;\;\;\;\;\;\;\;\;\;\;\;\;\mathrm{healthy}\;\mathrm{tissue}\;(k=2) \end{array}\right.$$

is volumetric heating rate dissipated by the volume fraction Φ of MNPs injected within tumoral tissue.

The **metabolic heat source, (**$Q_{met}^{k}\;k=1,\;2$**)**, represents the volumetric rate of heat generated by cellular metabolic activity within biological tissue and is expressed in W/m^3^. It corresponds to the endogenous heat production of the tissue and is distinct from the additional magnetic heat source generated by the magnetic nanoparticles during exposure to the alternating magnetic field. The values used in the simulations (Table 2), $Q_{met}^{1}$ = 31,872 W/m^3^ for tumoral tissue and $Q_{met}^{2}$ = 6374 W/m^3^ for healthy tissue, were considered from previously published bioheat modeling data [25].

### 3.2. Boundary Conditions

In this case, the following boundary conditions were used:

- Dirichlet-type condition on the outer surface of healthy tissue:

(20) $$T_{2}\left(r=R_{2}\right)=37\;{}^{\circ}C$$

- Neumann-type conditions at the tumor–healthy tissue interface:

(21) $$k_{1}{\left(\frac{\partial T_{1}}{\partial r}\right)}_{r=R_{1}}=k_{2}{\left(\frac{\partial T_{2}}{\partial r}\right)}_{r=R_{1}}\mathrm{and}\;T_{1}\left(r=R_{1}\right)=T_{2}\left(r=R_{1}\right)$$

Thermal damage is described by the following relation:

(22) $$u_{k}\left(r,t\right)\;=\;\int_{0}^{t}A_{k}\;exp\left(-\frac{E_{a}^{k}}{RT_{k}}\right)d\tau ,$$

where $E_{a}^{k}$ is the activation energy, $A_{k}$ is the frequency factor, R is the universal gas constant and τ is the tissue heating time. The expression $\gamma_{k}\left(r,t\right)\;=\;exp\left[-{\;u}_{k}\left(r,t\right)\right]$ defines the fractions of damaged tissues. Tissue temperature, $T_{k}$ strongly influences the evolution of this fraction of tissue damage.

### 3.3. Numerical Implementation

The computational domain was discretized using an **unstructured free tetrahedral mesh** generated automatically in COMSOL Multiphysics 6.2. The mesh was constructed with a maximum element size of **0.2**, a minimum element size of **0.036**, a maximum element growth rate of **1.5**, a curvature factor of **0.6**, and a narrow region resolution parameter of **0.5**. These settings provide an adequate balance between computational efficiency and numerical accuracy while ensuring a proper representation of the geometry and the physical fields involved in the simulation.

The numerical model consists of approximately **35,170 degrees of freedom (DOFs)**, indicating a sufficiently refined spatial discretization for resolving the coupled magnetic and thermal phenomena. The exact number of computational cells is determined by the generated tetrahedral mesh and can be obtained from the mesh statistics within COMSOL. The computational mesh was generated using the **free tetrahedral** meshing algorithm implemented in COMSOL. Mesh refinement was automatically controlled according to the specified element size limits and geometric features, allowing a finer discretization in regions with higher curvature and narrow domains where enhanced spatial resolution is required. The governing equations were spatially discretized using the **finite element method (FEM)** implemented in COMSOL Multiphysics. This discretization approach provides accurate numerical solutions for the coupled partial differential equations describing the magnetic and thermal processes. Time integration was performed using an **implicit backward differentiation formula (BDF)** time-dependent solver with adaptive time stepping and a maximum integration order of **2**. This implicit integration scheme is particularly suitable for solving the stiff nonlinear equations encountered in coupled magnetic and heat transfer simulations while maintaining numerical stability over the entire simulation interval. The nonlinear algebraic system was solved using a **fully coupled Newton solver**, whereas the linearized systems were solved using the **GMRES iterative solver**, configured with a maximum of **10,000 linear iterations**. The model also includes the **PARDISO direct solver**, which is available for selected solution stages when required. A damping factor of **0.9** was employed in the Newton iterations to improve the robustness of the nonlinear solution procedure.

The convergence of the numerical solution was monitored through the nonlinear residual, linear residual, and linear error during each time step. The adopted numerical strategy ensured stable convergence of the coupled multiphysics model and reliable prediction of the magnetic and thermal responses throughout the simulation.

## 4. Results and Discussion

The therapeutic temperature values and implicitly the thermal damage of a tumoral tissue are strongly dependent on the nanoparticle size $d_{p}$, magnetic field parameters, (i) frequency f(kHz) and (ii) field amplitude $H\left(\frac{kA}{m}\right)$, the volume fraction of nanoparticles injected into the tissue (Φ) and the interparticle surface-to-surface distance between nanoparticles α(nm). The magnetic field parameters used in the simulations satisfy the following clinical safety criterion: $H\;*\;f\;<\;5\;\cdot \;10^{9}\;A\;m^{-1}s^{-1}$, for magnetic hyperthermia treatments.

### 4.1. Dipolar Interactions and SAR

**Figure 3:**
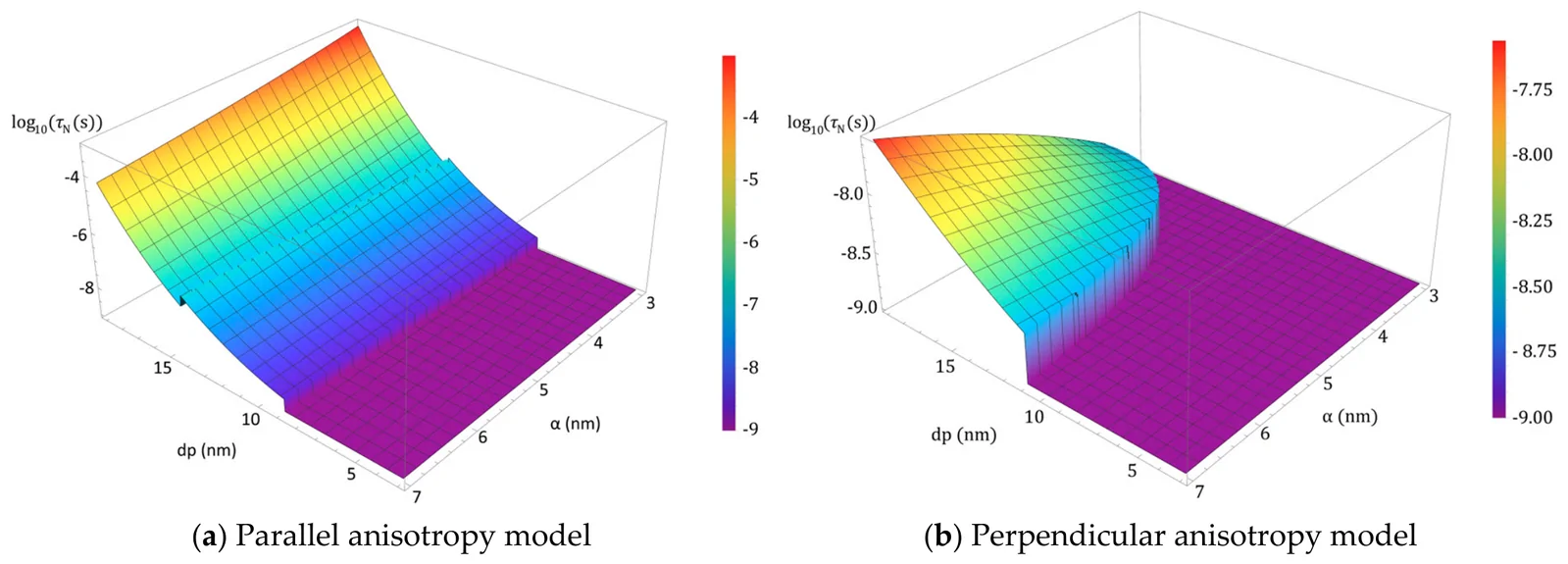
Logarithmic Néel relaxation time as a function of nanoparticle diameter and interparticle spacing for magnetite nanoparticles: (**a**) parallel anisotropy configuration and (**b**) perpendicular anisotropy configuration.

Figure 3a,b describe 3D logarithmic Néel relaxation time ($\tau_{N}$) in dependence with interparticle distance α(nm) and particle size ($d_{p}$) for the magnetite nanoparticles in the following cases: (i) the magnetic moments are oriented along the direction connecting the MNPs (**parallel anisotropy model**) (Figure 3a) and (ii) magnetic moments oriented perpendicular on the direction connecting the MNPs (**perpendicular anisotropy model**) (Figure 3b).

For small nanoparticles and large interparticle spacings α, magnetic dipolar interactions are weak, resulting in a relatively low effective energy barrier and fast Néel relaxation (Figure 3a). As the particle size increases and the interparticle spacing α decreases, dipolar coupling becomes stronger, modifying and, for the configuration considered here, increasing the effective energy barrier and consequently increasing the Néel relaxation time. The surface therefore illustrates the transition between three characteristic relaxation regimes: (i) regime I (σ < 1), corresponding to a low effective energy barrier and fast Néel relaxation; (ii) regime II (1 < σ < 5), an intermediate regime in which Néel and Brownian relaxation may become comparable; and (iii) regime III (σ > 5), characterized by a high effective energy barrier, strongly suppressed Néel relaxation, and an increasing relative contribution of Brownian relaxation as the particles approach the magnetically blocked regime.

The parallel anisotropy model (with increased effective energy barrier) predicts that stronger dipolar coupling appears for smaller interparticle spacing α (nm) and larger particle size and both increase the Néel relaxation time, indicating slower relaxation dynamics in strongly interacting chains.

Figure 3b shows the second dipolar configuration, in which the dipolar interaction reduces the effective energy barrier. For small nanoparticles, the Néel relaxation time remains very short and only weakly dependent on the interparticle spacing α, indicating fast magnetization reversal. As the particle diameter increases, τ_N_ increases markedly due to the increasing intrinsic anisotropy energy KV. However, decreasing the interparticle spacing α strengthens the dipolar interaction and, in this configuration, partially reduces the effective energy barrier, thereby counteracting the size-induced increase in τ_N_. The surface therefore reflects the competition between the intrinsic anisotropy energy, which increases strongly with particle volume, and the dipolar contribution, which lowers the effective barrier in this configuration.

**Figure 4:**
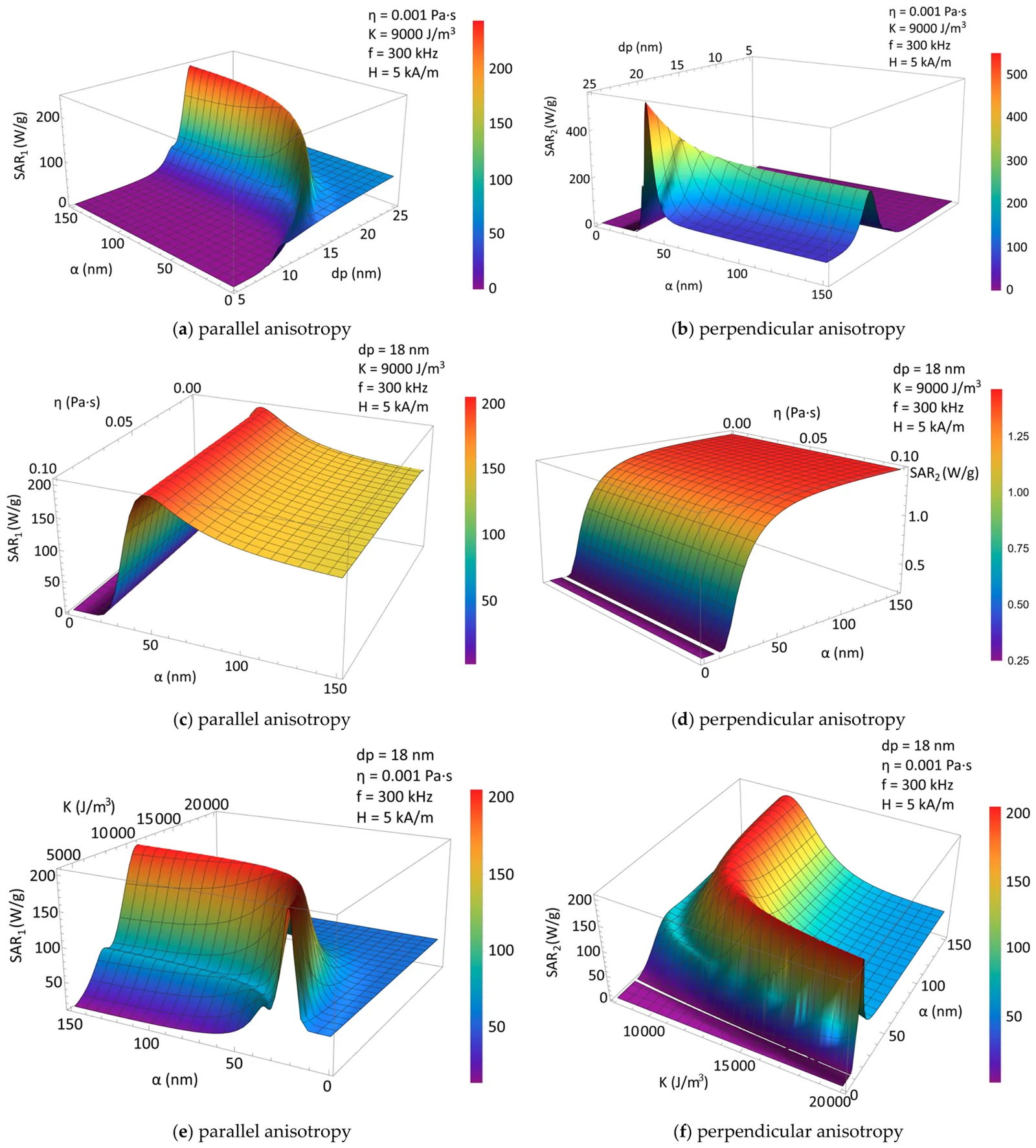
Effect of nanoparticle diameter d_p_, interparticle spacing α, viscosity η and magnetic anisotropy constant K on SAR for the two dipolar-interaction configurations: (i) parallel anisotropy (SAR_1_) and perpendicular anisotropy (SAR_2_).

**Effect of nanoparticle diameter and interparticle spacing** α **on SAR.** Figure 4a shows the variation of the specific absorption rate ${SAR}_{1}\;(W/g)$ with size (diameter) ($d_{p}$) and interparticle spacing in the parallel anisotropy model for an alternating magnetic field applied with the following parameters: H = 5 kA/m and f = 300 kHz. The size of the nanoparticle fundamentally influences the increase in the ${SAR}_{1}$ parameter. An increase in SAR values is observed with the diameter of the MNPs, reaching a maximum in the range of 12–18 nm, due to the significant influence of anisotropy energy in the Néel relaxation processes. Then, SAR values decrease significantly, indicating the transition from an optimal relaxation regime to a regime with more stable anisotropy barriers in which magnetic moment relaxation becomes slower. The energy dissipation process is influenced by the parameter α(nm) associated with the distance between nanoparticles. Dipolar interaction increases when particles are close together (low α values), contributing to the increase in the effective anisotropy barrier. In this case, the slower relaxation of the magnetic moment causes a decrease in SAR values. As α(nm) increases, the particles are more dispersed, the dipole interaction decreases, and the lower effective anisotropy barriers favor faster relaxation of the magnetic moment, causing an increase in SAR values.

Figure 4b describes the variation of the specific absorption rate ${SAR}_{2}\;(W/g)$ with size (diameter) ($d_{p}$) and distance between MNPs in the perpendicular anisotropy model for an alternating magnetic field applied with the same parameters: H = 5 kA/m and f = 300 kHz. The maximum SAR values and consequently an optimal energy dissipation are observed in the size range 18–20 nm, where both relaxation mechanisms are activated. At very small MNP sizes (below 10 nm), SAR values are reduced because the magnetic moment fluctuates thermally and Brownian relaxation is negligible. As the diameter increases, the coupling with the viscous medium becomes more efficient and the rotation of the particles in the fluid amplifies the dissipated energy.

Interparticle distance α(nm) also influences SAR values in this case. For small α(nm) distances, the particles are close together and strong dipole interactions reduce the anisotropy barrier, causing the magnetic moment to relax too quickly. As a result, the decrease in magnetic moment relaxation efficiency leads to a decrease in SAR values.

The increase in the distance between particles α(nm) causes a significant reduction in dipole interactions, the effective energy barrier is reduced to the initial KV value, and the two relaxation mechanisms become comparable, causing an increase in SAR parameter values. Figure 4b highlights the optimal range of MNP size and interparticle distance in which SAR values are maximum. In practice, strong dipole interactions (which occur at small distances between particles) reduce the efficiency of magnetic hyperthermia, while weak and/or moderate interactions (which occur at greater distances) increase the efficiency of magnetic hyperthermia by optimizing SAR values.

**Effect of viscosity and interparticle spacing on SAR**. Figure 4c demonstrates the independence of SAR values from ferrofluid viscosity (the Brownian relaxation process is almost negligible), confirming the dominance of Néel relaxation. It can also be observed that SAR values are significantly dependent on interparticle distance. Figure 4d shows that SAR values decrease slightly with increasing viscosity of the medium. In other words, nanoparticles heat more efficiently in media with lower viscosity.

**Effect of magnetic anisotropy and interparticle spacing on SAR. **Figure 4e shows the dependence of SAR values on interparticle distance α(nm) and anisotropy constant K for nanoparticles with a diameter of 18 nm. SAR values increase with the distance between nanoparticles and show a maximum for intermediate values of anisotropy K. For higher values of K, the magnetic moment relaxes over a longer time, which causes SAR values to decrease. Figure 4f highlights a pronounced maximum depending on the anisotropy constant. At low values of K, the energy barrier is insufficient to stabilize the magnetic moment, which results in low SAR values and therefore low heating. SAR reaches a maximum for intermediate values of K where Néel relaxation is optimal. For high values of K, the magnetic moment becomes locked and, as a result, SAR values decrease significantly. SAR values are higher for dispersed MNPs (high values α(nm)). Strong dipole interactions at short distances reduce the effective energy barrier, which destabilizes the orientation of the magnetic moments and results in low SAR values (the magnetic moments relax very quickly). As α(nm) increases, the dipole coupling becomes very weak and the KV anisotropy energy becomes dominant. In this case, Néel-type relaxation dominates, causing SAR to increase to a saturated value for very large distances between particles. This effect may be present in dilute colloidal suspensions.

The relative importance of the Néel and Brownian mechanisms can be assessed by comparing their characteristic relaxation times. Since the effective relaxation time is defined as

$$\tau_{i}=\frac{{\left(\tau_{N}\right)}_{i}\;\tau_{B}}{{\left(\tau_{N}\right)}_{i}+\tau_{B}}$$

the faster mechanism predominantly controls the magnetic response. Thus, when effective relaxation time approaches ${\left(\tau_{N}\right)}_{i}$, Néel relaxation dominates, whereas for ${\left(\tau_{N}\right)}_{i}\;\gg \;\tau_{B}$ effective relaxation time approaches $\tau_{B}$ and Brownian relaxation becomes dominant. Figure 4c shows that SAR is nearly independent of the ferrofluid viscosity over the investigated range, indicating that ${\left(\tau_{N}\right)}_{i}$ is the relevant timescale and that the heating response is predominantly Néel-controlled. In contrast, the slight decrease in SAR with increasing viscosity observed in Figure 4d indicates a larger, although still secondary, influence of Brownian relaxation. Therefore, under the conditions corresponding to the main SAR maxima investigated here, Néel relaxation provides the dominant contribution to the effective magnetic relaxation, while Brownian rotation acts mainly as a secondary mechanism whose influence increases when the two characteristic relax times become comparable.

In the present model, the term moderate dipolar interaction refers to an intermediate coupling regime in which the dipolar contribution modifies the effective energy barrier while maintaining the relaxation dynamics close to the optimal condition $\left(2\pi f\right)\tau_{i}\;\approx 1$. For nanoparticles within the optimal diameter range 12–20 nm, the calculated SAR maps indicate that the region around α ≈ 30–50 nm represents the transition from strong to moderate dipolar coupling. At larger interparticle separations, the dipolar contribution rapidly decreases according to ${\left(d_{p}\;+\;\alpha \right)}^{-3}$, and the system progressively approaches the weakly interacting limit.

**Figure 5:**
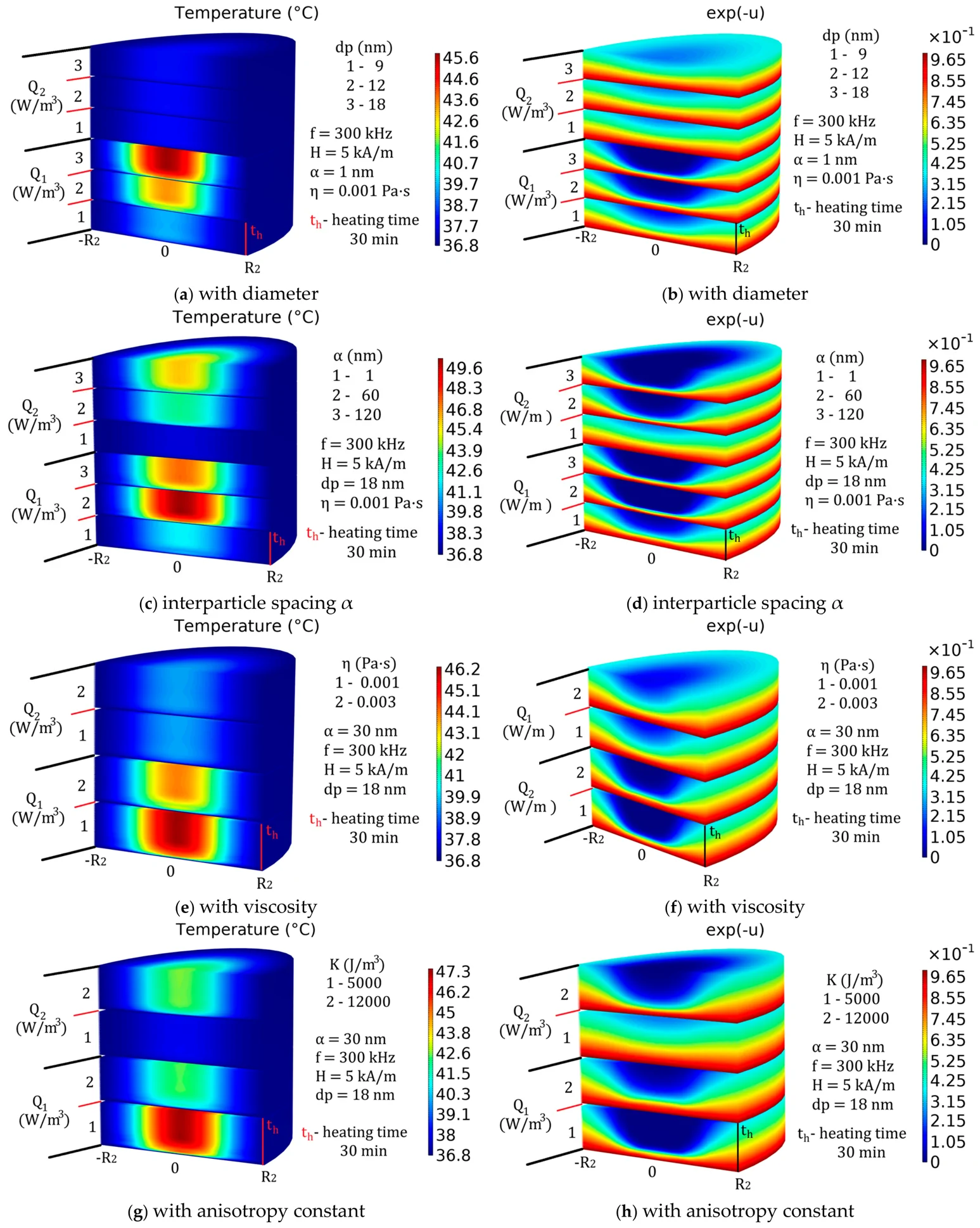
Effect of nanoparticle diameter d_p_, interparticle spacing α, carrier-medium viscosity η, and magnetic anisotropy constant K on tissue temperature and thermal damage.

Figure 4 and Figure 5 represent two successive levels of the computational framework. Figure 4 describes the nanoparticle-scale magnetic response and the resulting SAR, whereas Figure 5 shows the corresponding tissue-scale thermal response obtained by introducing the magnetic power dissipation as a volumetric heat source into the 3D bioheat model.

### 4.2. Thermal Response and Thermal Damage

Figure 5 describes the spatial–time evolution of the temperature distribution and the corresponding thermal damage predicted by the bioheat model for different nanoparticle parameters. The thermal response follows the SAR behavior previously presented in Figure 4, confirming that the magnetic heating efficiency directly determines the temperature increase within the tumoral tissue.

**Figure 5a,b** show the influence of nanoparticle diameter on tissue temperature and thermal damage. The highest temperatures are obtained for nanoparticle diameters corresponding to the maximum SAR values identified in Figure 4. Consequently, the Arrhenius thermal damage parameter reaches its highest values in the same diameter range, indicating more effective destruction of the tumoral tissue. Outside this optimum interval, the reduced heating efficiency leads to lower tissue temperatures and less pronounced thermal damage.

**Figure 5c,d** present the effect of interparticle spacing. The temperature distribution closely follows the SAR dependence on dipolar interactions discussed previously. Moderate interparticle spacing, corresponding to optimal magnetic losses, produces the highest temperature increase and the largest damaged region. At very small interparticle distances, strong dipolar coupling reduces the heating efficiency, whereas for sufficiently large separations the nanoparticles behave almost independently and the thermal response approaches that of noninteracting particles.

**Figure 5e,f** illustrate the influence of ferrofluid viscosity. Lower viscosities promote more efficient magnetic relaxation and therefore higher heat generation, resulting in increased tissue temperatures and larger thermal damage. As the viscosity increases, the thermal response becomes progressively weaker, consistent with the reduction in SAR observed in Figure 4.

**Figure 5g,h** show the dependence on the magnetic anisotropy constant. The maximum temperature rise and thermal damage are obtained for intermediate anisotropy values, where the magnetic relaxation process is most efficient. Both lower and higher anisotropy constants reduce the heating efficiency, leading to smaller temperature elevations and less extensive tissue damage.

Overall, Figure 5 demonstrates that the temperature field and the resulting thermal damage are direct consequences of the magnetic power dissipated by the nanoparticles. Therefore, the thermal behavior predicted by the bioheat model consistently reflects the SAR variations determined by nanoparticle diameter, interparticle spacing, ferrofluid viscosity, and magnetic anisotropy.

### 4.3. Comparison with Experimental Data

The calculated SAR values can also be compared with experimental magnetic hyperthermia data reported for iron-oxide nanoparticles. Under the conditions considered in the present study, H = 5 kA/m and f = 300 kHz, the predicted maximum SAR values are of the order of (10^2^) W/g, with optimal nanoparticle diameters of approximately 12–18 nm for the parallel configuration and 18–20 nm for the perpendicular configuration. Experimental studies have similarly reported SAR/SLP values ranging from several tens to several hundreds of W/g for iron-oxide nanoparticles, depending strongly on particle size and magnetic-field conditions [26]. Moreover, Branquinho et al. [9] showed that dipolar interactions and chain formation significantly modify the heating efficiency, with the resulting SLP depending on particle size, interparticle separation, chain organization, and polydispersity. Although a direct quantitative comparison is limited by differences in nanoparticle properties and experimental field conditions, the order of magnitude of the calculated SAR values, the predicted optimal size range, and the dependence on dipolar interactions are consistent with published experimental observations [9,26].

### 4.4. Model Limitations

The present analytical model intentionally considers a sufficiently long one-dimensional chain of identical single-domain nanoparticles with perfectly aligned easy axes and two limiting anisotropy configurations (parallel and perpendicular). Structural disorder, particle-size distributions, random anisotropy orientations, finite-chain effects, and three-dimensional aggregation are not included. These factors may broaden the effective energy-barrier and relaxation-time distributions in realistic nanoparticle assemblies. The present simplified model is intended to isolate **the fundamental role of dipolar interactions** and provide a **transparent framework for evaluating their influence on magnetic relaxation, SAR, and tissue heating**.

## 5. Conclusions

This study analyses the influence of dipole–dipole interactions on the heating efficiency of MNPs for magnetic hyperthermia. A dipolar coupling in a chain of MNPs was considered to evaluate the effective anisotropy barrier and its impact on the Néel–Brown relaxation mechanisms.

Dipolar interactions significantly modify the effective energy barrier governing magnetization reversal. Depending on the orientation of the magnetic moments relative to the interparticle axis, dipolar coupling may either increase or decrease the effective anisotropy barrier. These two cases correspond to the parallel and perpendicular interaction models, which represent two limiting regimes of dipolar coupling in interacting nanoparticles.

Three characteristic relaxation regimes were determined by the competition between thermal energy and the effective anisotropy barrier: (i) fast superparamagnetic relaxation, (ii) an intermediate Néel–Brown regime, and (iii) a slow blocked regime. Nanoparticle size and interparticle spacing are two very important parameters in the magnetic relaxation processes and heating efficiency in nanoparticle assemblies.

Optimal magnetic heating is obtained for intermediate nanoparticle diameters and moderate dipolar interactions, where the magnetic relaxation time becomes comparable to the period of the applied alternating magnetic field. Strong dipolar interactions at very small interparticle distances may lead to magnetic locking and reduced heating efficiency.

The model therefore provides useful guidelines for the optimization of nanoparticle parameters in magnetic hyperthermia applications.

## Figures and Tables

**Table 1:** The main parameters used in simulations.

Symbol	Definition	Units
K	Magnetic anisotropy constant	J/m^3^
M_s_	Saturation magnetization	A/m
V	Nanoparticle volume	m^3^
d_p_	Particle diameter	nm
α	Surface-to-surface interparticle spacing	nm
r	Center-to-center distance	nm
m	Magnetic moment	A·m^2^
$\tau_{N}$	Néel relaxation time	s
$\tau_{B}$	Brownian relaxation time	s
τ	Effective relaxation time	s
SAR	Specific absorption rate	W/g
$\tau_{0}$	Average relaxation time	s
δ	Surfactant layer thickness	nm
η	Carrier liquid viscosity	Pa·s
V_H_	Hydrodynamic volume of the particle	m^3^
f	Magnetic field frequency	kHz
H	Magnetic field amplitude	kA/m
ρ	Magnetic material density	kg/m^3^
P_i_	Volumetric power loss	W/m^3^
Φ	Volume fraction of the magnetic nanoparticles	dimensionless
Q	Volumetric heat source	W/m^3^

The table describes the main parameters used in this paper.

**Table 2:** Thermophysical properties of the tumoral and healthy tissues used in the bioheat model.

Property	Tumor Tissue (*k* = 1)	Healthy Tissue (*k* = 2)
Density $\rho_{k}$ (kg/m^3^)	1045	1045
Specific heat, $c_{k}$ (J/(kg·K))	3760	3760
Thermal conductivity, $k_{k}\;(W/(m$·K))	0.56	0.50
Blood perfusion rate, ${\omega_{b}}^{k}$ (s^−1^)	0.0095	0.0036
Blood density, $\rho_{b}$ (kg/m^3^)	1060	1060
Blood specific heat, $c_{b}$ (J/(kg·K))	3770	3770
Blood temperature, $T_{a}$ (K)	310.15	310.15
Metabolic heat source, $Q_{met}^{k}$ (W/m^3^) [25]	31,872	6374

## Data Availability

The authors declare that all data supporting the findings of this study are available within the article.
